# Supplementary material for: Chromothripsis during telomere crisis is independent of NHEJ, and consistent with a replicative origin
Source: Genome Res. 2019 May;29(5):737–49. doi: 10.1101/gr.240705.118 (PMC6499312; doi:10.1101/gr.240705.118)
Supplement: Supplemental Material [file supp_gr.240705.118_Supplemental_file_1.zip › contigs/annotated_contigs/DB81/contig.2.DB81_length_646_mean_cov_6.96594427245.docx]

**DB81_length_646_mean_cov_6.96594427245**

```
AAGGGAGGGGCAGGCTGCTCG|AGCTTCGTGTTTAATTGCTTCAAGGGGAGGGGCCCTGGGGAAACAATTTTCTGGGAATTTACAAAGA
                      >chr3:51703182-51703549 + E=5e-188
AGGATGGAGAAAGGATCTCTGGAGTGAGTCCTTGGACAGAGGAAGGCAAGAGGAGATGAAGGCCAAGTCTGGACTCATGATAGGGGAAT

CTGTGCTATTGGGAGATAAGATTGGGAGTGGAGGATGGTTGCAGCACATGGCTGGGCGAACAGGTTTCCTCAGACCTGTTACAAGGGGT

TGGAATGAAGAAAGTGCCTTGTAGAGTAGGCTGCTGCCTCCTGGTGGCCAGCCGCTGCCTTGCAGGGATCCTGCCAAGGGACCTCACTT

GGCACTGTTGGAAGCAGCCTGAGGTGAAGCTGG|AAGGGCACTTTTGAAAAATGAA|TGTGGTCATTTGGCTGGCATGACCACAGCCTC
                                                        >chr3:51712718-51712954 + E=3e-
ACAGAGGCTGGTGAGAGTTCTGATGTAGGCCTGTGTCACACTCCCTCCTCCCTCAGTGTGTGTGGAGAGAAGGCTCCCTGCCCTCCCTG
130
GAGGCACTTAGTTTGGGTGGAGCCTGGGAAGGGGTGAGGGAGTAGGATTTCCCAGGACTGACCTCCTCAACAAAGGCAATGGGAATGTG

GGCTGGGATGTTTCCTGAGGGCTCAG
```
